# Lived Experiences of Suicide Risk and Resilience among Alaska Native and American Indian People

**DOI:** 10.3390/ijerph16203953

**Published:** 2019-10-17

**Authors:** Jennifer L. Shaw, Julie A. Beans, Katherine Anne Comtois, Vanessa Y. Hiratsuka

**Affiliations:** 1 Research Department, Southcentral Foundation, Anchorage, AK 99508, USA; jshaw@scf.cc (J.L.S.); vhiratsuka@scf.cc (V.Y.H.); 2 Department of Psychiatry and Behavioral Sciences, Harborview Medical Center, University of Washington, 325 9th Ave, Box 359911, Seattle, WA 98104, USA; kcomtois@uw.edu

**Keywords:** Alaska Native, American Indian, lived experience, suicide

## Abstract

This study explored the lived experiences of suicidality and help-seeking for suicide prevention among Alaska Native and American Indian (AN/AI) people in a tribal health system. An interpretive phenomenological approach was used to analyze semi-structured, in-depth interviews with 15 individuals (ages 15–56) with self-reported histories of suicide ideation and/or attempt. Several factors were found to be central to acquiring resilience to suicide risk among AN/AI people across a wide age range: meaningful and consistent social connection, awareness about how one’s suicide would negatively effect loved ones, and knowledge and utilization of available health services. Findings highlight the mutable nature of suicide risk and resilience, as well as the importance of interpersonal factors in suicidality.

## 1. Introduction

Suicide is a major public health concern for Alaska Native and American Indian (AN/AI) people in the United States (US). Although the age-adjusted suicide rate for AN/AI people is consistently less than that of the general US population (13 versus 14/100,000), the suicide rate among the AN/AI population has increased faster than in the general US population since 2000 (28% versus 25%, respectively) [1]. Suicide-related disparities are particularly striking in some geographic regions. In Alaska, suicide among AN/AI (all ages) is consistently three to four times higher than the US rate [2] and is the leading cause of death among those age 10–64 years [3]. The suicide rate in Alaska was either the first or second highest in the nation from 2012–2017 [3]. Suicide occurred in higher rates among males, AN/AI people, persons aged 20–24 years, and among those in rural areas. However, rates increased in urban areas during 2012–2017 [3]. Furthermore, suicide deaths for AN/AI people may be underestimated by up to 33% due to misclassified race and ethnicity groups [4]. Additionally, for every suicide, there are an estimated 25 suicide attempts and many more cases of suicide ideation [5].

Numerous studies have examined risk factors for suicide in AN/AI communities. These investigations primarily focused on individual risk factors for AN/AI suicide, such as depression, trauma (e.g., child abuse), and substance abuse [6,7,8,9,10,11]. In the last decade, studies have increasingly focused on social and structural sources of AN/AI suicide risk, including colonization and its sequalae (e.g., social disruption, intergenerational trauma) [12,13,14,15]. There is now a robust corpus of evidence on multi-level risk factors for suicide in AN/AI communities, including individual, social, and structural factors. A few studies have looked at what protects AN/AI individuals and communities from suicide risk. Factors posited to reduce suicide risk in AN/AI communities include: cultural continuity, community safety, cultural and social connectedness, female community leaders, and self-determination [16,17,18,19,20,21]. Allen et al. (2018) developed and are evaluating a community-level intervention to promote resilience and reduce suicide risk in AN communities through an intervention that uses “culture as intervention”, where Yup’ik values and ways of being and knowing contribute to reasons for life and sobriety [22]. However, little empirical evidence exists for interventions that foster resilience among AN/AI individuals already experiencing suicide risk. Culturally appropriate, evidence-based interventions are needed to promote AN/AI resilience to suicide risk at the individual level [23,24,25]. Such interventions may be a more fruitful approach than those aimed at reducing risk factors, such as trauma, which may have already occurred [16]. 

Until recently, little was known about the lived experience of individuals of any background with histories of suicidality, either ideation or attempts. Cutliffe and Cummins (2004) point out that understanding these experiences and the meanings that individuals attach to them is critical for developing effective interventions at all levels of care—preprimary, primary, and secondary [26]. Understanding lived experience elucidates not only what it means to be suicidal, but also how people survive this potentially deadly experience. To date, we have found no studies that have explored the lived experience of AN/AIs who have sought help for suicide ideation and attempts. Further, few studies have investigated suicidality in AN/AI individuals living in an urban setting [16]. The lived experiences of AN/AIs who have sought help for suicidality may offer valuable guidance for researchers to develop and test interventions to increase resilience to suicidality among those at highest risk. This guidance could also prove useful to clinicians and health systems caring for AN/AI who are at risk of suicide [27]. 

Unlike epidemiological studies of suicide risk, qualitative case studies can help to explain the social and cultural context of suicidality among AN/AI people and how those affected by suicidality make meaning of these experiences. Understanding the subjective, lived experiences of those affected by suicidality has important potential to inform the innovation of culturally appropriate health services, shape public policy, and promote meaningful social change [28]. This paper reports on an interpretive phenomenological qualitative study that explored the lived experiences of AN/AI individuals who have sought help for suicidality, with particular attention to factors that helped them to stay alive.

## 2. Materials and Methods

### 2.1. Design and Aims

This study used semi-structured interviews to explore the lived experience of AN/AI individuals who sought help for suicidality. We used interpretive phenomenological analysis (IPA) to examine: (1) the experiences that participants described as contributing to their suicide risk; and (2) the experiences that they perceived to have reduced their suicidality. IPA is a qualitative methodology used to examine and understand the subjective, lived experience of individuals using an idiographic approach, in which detailed, individual cases are used to develop more general claims [29]. As Smith and Osborn [29] point out, IPA is “especially valuable when examining topics which are complex, ambiguous and emotionally laden”, such as suicide. IPA has been used in previous studies to understand the experiences of health care providers who care for suicidal people [30], the experiences of family members and friends of suicidal individuals [31], and the experiences of suicidal individuals themselves [32,33]. 

### 2.2. Setting

The study was conducted at Southcentral Foundation (SCF), an Alaska Native-owned and operated health care organization offering primary care, dental, and behavioral health services for 65,000 AN/AI people in the mostly urban Anchorage and Matanuska-Susitna Boroughs and 55 rural villages [34,35]. Suicide prevention occurs at multiple points in the health care system. In primary care, licensed, behavioral health professionals provide brief intervention and referral to longer-term specialty behavioral health care, such as medication management, counseling, or case management. The SCF Behavioral Urgent Response Team (BURT) provides crisis intervention and assessment in the Alaska Native Medical Center, as well as short-term medication management, and refers patients to outpatient, specialty behavioral health services, a sub-acute, residential crisis recovery center, an acute, short-term, inpatient psychiatric unit, or the state psychiatric hospital. In addition, all primary care staff take a half-day course called SafeTALK [36], and SCF offers Applied Suicide Intervention Skills Training [37] to all employees and patients, free of charge.

### 2.3. Sample

Participant inclusion criteria were: (1) self-identified AN/AI, (2) 15 years of age or older, who (3) spoke English, (4) were living in the SCF service area, and (5) had a self-reported history of seeking help for suicidality. Individuals who reported having suicide ideation and/or attempts within the previous six months were excluded from the sample for participant safety. Help was defined as psychological or medical support, care, or treatment provided by someone with whom the person had a formal (e.g., health care provider) or informal (e.g., family member) relationship. We initially aimed for a sample of 10 people; however, due to interest in the study and the need to reach data saturation, we expanded the sample size to 15. 

### 2.4. Recruitment

Recruitment occurred between January 2013 and May 2015 through fliers posted in the SCF clinic waiting rooms and lobbies. Fliers provided information about the study and a phone number for the SCF Research Department. Interested individuals who called to inquire about the study were transferred to the principal investigator (J.L.S.) and informed that they would need to answer several eligibility questions, including whether they had ever personally sought help for suicidal thoughts or behavior. If they agreed to proceed, they were screened and scheduled for an interview if eligible.

### 2.5. Informed Consent

An informed consent document provided to each participant explained the purpose of the research, detailed potential risks, and provided a confidentiality statement of how participant information would be securely handled. In addition, the informed consent document acknowledged that there was likelihood that the participant could feel emotional discomfort during the interview and noted that the researcher could provide a list of available suicide prevention services; or, if the participant needed to speak with a professional immediately, the researcher could connect the participant to an available SCF behavioral health professional. The researcher reviewed the informed consent with each participant and offered time for questions. All participants provided written consent and were provided a copy of the informed consent to take with them. Written child assent and parental permission were obtained for participants under 18 years of age.

### 2.6. Data Collection

Fifteen semi-structured, individual interviews were conducted by a researcher (J.L.S.) trained in qualitative methods. The interviews were conducted in SCF’s Anchorage Native Primary Care Center, which is a familiar setting for participants, where SCF behavior consultants were in close proximity in the event that a participant needed immediate support. An interview guide with open-ended questions was used to elicit the individual’s experience with suicidal ideation and suicide attempts, experiences of seeking and receiving help, and perceptions of how these experiences affected their health and well-being. Due to the small sample size, we chose not to collect social, education, and employment background information to protect participant privacy. All interviews were audio recorded and transcribed verbatim. Interview recordings and transcribed interviews were saved on a password-protected computer and stored on a secured network server to maintain confidentiality. Interviews lasted 90 minutes on average. Each participant received a $50 gift card. All study procedures were approved by the Indian Health Service’s Alaska Area Institutional Review Board (#2012-08-031 approved 19 November 2012), and community-level approval was obtained from the SCF Research Review committees [38].

### 2.7. Data Analysis

We used an interpretive phenomenological approach to analyze these data [39,40,41]. A small sample size is required for IPA, which allowed us to explore participants’ experiences of surviving suicidality in greater depth than a thematic approach would have. Transcripts were independently reviewed by two qualitative researchers trained in medical anthropology (J.L.S.) and public health (J.A.B.). Each researcher developed in-depth summaries of each transcript. The researchers then met to discuss these summaries and develop a code list together to ensure intercoder reliability. Once consensus was reached, the code list was then applied to each transcript. Coded data were examined to characterize experiences of suicidality and related help-seeking, identify patterns in these experiences, and describe key protective factors. Interrater reliability was achieved through iterative meetings in which the analysts discussed the data until consensus was reached on the final results.

## 3. Results

### 3.1. Participant Characteristics

#### 3.1.1. Demographic Characteristics

The sample consisted of 15 AN/AI participants between the ages of 15 and 56 years, nine of whom were female. While all lived in the urban center of Anchorage, Alaska at the time they were interviewed, 11 participants grew up in rural Alaska communities or out of state. 

#### 3.1.2. Histories of Suicidal Thoughts and Behavior

The age at which participants first experienced a suicidal thought or behavior ranged from 9 to 35 years. Participants’ mean age of first suicide ideation or behavior was 17 years. Two-thirds of participants experienced suicidal ideation or behavior by age 15. These experiences occurred as early as the 1970s and up to six months prior to the interview date. Six participants experienced suicidal ideation only. Nine participants had attempted suicide, all on multiple occasions. Participants with no history of suicide attempt had sought and received help that they perceived to be effective from a family member or health care professional within days or weeks of having their first thoughts of suicide. Some participants sought help from informal sources only, such as family members, colleagues, or community members; others solely sought help from formal sources such as the health care system or the suicide support line. Most participants who had attempted suicide did not seek help prior to their first attempt, and some did not seek help prior to their second or third attempt. All participants indicated that eventually, if not at first, the help they received did decrease their suicidality. Some participants experienced ongoing or chronic suicidality for many years after receiving help, and continued to struggle with suicidal ideation. 

### 3.2. What Put Participants at Risk

Experiences that contributed to suicide risk varied across individuals, but predominantly involved at least one, but usually several, of the following factors: (1) trauma and related health problems, (2) loss and exposure to suicide, (3) substance misuse, (4) lack of resources, and (5) stigma. 

#### 3.2.1. Trauma and Related Health Problems

All but one participant recalled traumatic experiences in childhood, including abuse (sexual, physical, emotional), witnessing domestic violence, and loss (parent or guardian death or abandonment). One participant was sexually abused as a child and later sexually abused another person, for which he spent time in prison. Another participant recalled that her first memory, at the age of three, was of being abused. As children, several participants lived in homes with chronic alcohol or other substance misuse. Most had experienced multiple traumas in their lifetimes. Participants described suicidality as one of the lasting and most profound impacts of their traumatic experiences.

#### 3.2.2. Loss and Exposure to Suicide

Participants had experienced a great deal of loss in their lives. Some participants experienced, as children, the death or abandonment by one or both parents. Most participants had also experienced the death of at least one loved one by suicide, including first- and second-degree relatives and close friends. Some had lost more people to suicide than they could remember. One participant noted that when she was growing up, suicide was so common that “we thought it was normal”. Another said, “Suicide really runs in our family pretty high.”

#### 3.2.3. Substance Misuse

Substance misuse was a common theme throughout participants’ narratives. The majority of participants grew up in households with substance misuse or had developed addiction themselves. Two lost parents due to alcohol-related injury or disease, and two spoke about parents still struggling with addiction. Eleven participants faced their own struggles with addiction. Three experienced dependence on alcohol or other drugs by adulthood, and one was homeless for two years due to a polysubstance addiction. Participants associated substance misuse with their worst life experiences, as reflected in the comments of one woman who described it this way: “Failure. In a lot of stuff. Like going to [juvenile] jail. Not finishing up school in the beginning. Going to adult jail. Losing my child. I mean, all these–looking back, everything is alcohol-related.” Using alcohol or drugs, being in environments where others are using alcohol or drugs, and even the smell of alcohol was described by several participants as a trigger for suicidal thoughts and behavior. One participant recounted, “Alcohol really messed my brain up, the thinking part. And abusing the alcohol instead of getting help, it made the depression worse and the suicidal thoughts worse.”

#### 3.2.4. Lack Effective Behavioral Health Resources

Another contributing factor to suicide risk described by participants was a lack of behavioral health resources to address anxiety, depression, and seasonal affective disorder. Resources were lacking because they did not exist, were not accessible, or were ineffective. A woman who initially experienced suicidal ideation as a teen in the 1970s recalled that services were not available in her community at that time. The lack of effective behavioral health services was described by several participants. One participant recalled telling someone that she was “tired of living” and was then admitted to a hospital where no one asked her about the reasons for her suicide ideation, which included sexual and physical abuse at home. She described being discharged and returning home, where the abuse continued, and she attempted suicide the following year. Upon calling a crisis intervention hotline, one participant was asked if she was going kill herself right then and was told to “call back when I was really suicidal”. 

#### 3.2.5. Stigma Related to Suicidality and Behavioral Health Care

Finally, primary risk factors for suicidality, such as trauma and substance misuse, were compounded by the social stigma participants experienced related to suicide and seeking behavioral health care. Stigma prevented nearly all participants from seeking help at some point in time. One participant said that, “If those [suicidal] thoughts were not a shockingly big deal, then there would be a lot more safe people out there.” Stigma prevented this participant from seeking help from teachers or other trusted adults as a suicidal teenager. A second participant recounted feeling ashamed when hearing health professionals speak about her in an emergency room during a suicidal crisis. Another participant, whose son had died by suicide in his 20s, suggested that men, in particular, would be more willing to seek help if suicide prevention services were integrated into general health care settings, such as primary care clinics, so that it is not obvious that someone is seeking behavioral health care. Finally, one participant said that “for a Native person, it is very impolite to speak about yourself”, and suggested that more people would disclose suicidal thoughts in emergency rooms if questioned in more culturally sensitive ways, such as saying, “We have a [counselor] available. Would you like to talk with them?”

### 3.3. What Kept People Safe

Participants also recounted stories of staying safe during times when they had experienced thoughts of suicide. These stories were characterized by a multitude of diverse resources that are described below in three, broad categories: (1) informal support, (2) formal support, and (3) self-support. 

#### 3.3.1. Informal Support

Participants described a wide array of caring people who entered and/or became active in their lives at key moments in time. One participant recalled a brief impactful moment with a police officer, “A cop arrested me… I asked [him], ‘How come you don’t let me die in my house with my drugs and alcohol?’ He says, ‘…I really care about you. You got to do something about your problems. Find the solution, accept the solution’. I was in denial for 30 years… and the short cut was to surrender [to] my problems… Like throw the white flag and accept, ‘What do I do now? How could I change… and get healthier or better for myself?’ That was the hardest thing.”

Several participants credited family members and friends with keeping them safe, such as one participant who became suicidal as a young man after his best friend died by suicide. His friend’s mother recognized the warning signs and arranged for a friend to stay with him until he was no longer contemplating suicide. Others described spiritual mentors as providing critical social support.

#### 3.3.2. Formal Support

Participants recounted numerous experiences with health care services that helped to keep them safe during difficult periods. These included: crisis intervention, specialty case management, individual and group therapy, medication management, residential and outpatient substance use treatment, primary care, and youth programming. 

Seven participants recounted helpful experiences with crisis intervention services, specifically within the tribal health system. One participant in her 40s with a history of multiple suicide attempts said, “I’m glad you guys have the BURT [crisis intervention] team, always keep that. That saved me so much.” A man in his 40s said the Alaska Native Medical Center emergency department was helpful to him because it is specifically for AN/AI people. Another participant said, “Sometimes I’ll find myself here at the behavioral health here in the hospital…and sometimes I don’t even know how I got here. And this is my safety place right here.”

In addition to the in-person crisis services, several participants relied on the Careline, Alaska’s statewide crisis hotline. A participant in her 50s said that, after several suicide attempts and psychiatric hospitalizations, she saw advertisements for the Careline on buses and began using it when she was suicidal. 

Four participants received services through Denaa’ Yeets, SCF’s suicide prevention program. For some, this specialized professional help was preferable to other types of support. One participant said, of talking with program staff, “It [suicide] was always in mind. It’s really hard to get away from it. But… if I talk to people that I can trust like Denaa Yeets’ or you, it helps me not to hurt myself. It keeps me in focus. I don’t like to show it to any other people because I know they’ll use that against me and…some of the things I bring up, it might be too harsh for them.”

In addition to checking in regularly by phone with people enrolled in the service, Denaa’ Yeets case managers organize group activities and outings such as berry picking or traditional Alaska Native sewing, and sometimes provided transportation. One participant in her 50s spoke about the program’s role in keeping her safe and socially engaged: “I like that, for them to do stuff like that for mostly people that have bad PTSD to get them out like that so that they can try to feel comfortable out in the world again. I like that because I would have been stuck indoors and not wanting to get out.”

Several participants talked about individual therapy as having helped them at times when they were vulnerable to suicidal thoughts or behaviors. Three themes ran throughout these stories. First, some participants described individual therapy as a place where they felt cared about. “She [the counselor] connected with me right away and I felt like she cared. And that was a time in my life where I needed somebody to care.” The second theme was feeling understood. One participant described feeling understood by her therapist, “…the way she showed that she was concerned for me was something that I didn’t have for a quite a while. Somebody cares; somebody cares about me. ‘Wow, you’re actually listening to what I’m saying, and you’re understanding what I’m going through?’ I thought nobody could do that. Because that was the part of the depression for me, like nobody can understand the depths of what I’m going through. And for the first time in a long time I felt like she did.” Finally, gaining useful skills was the third theme. Therapy provided practical skills that helped participants stay alive. For instance, a man in 30s described his third experience with therapy as a success, “It gave me more tools that I could add to my tool bag, just to get through life.” In addition, several participants found that medication to treat insomnia, anxiety, and depression had helped them. However, medication was not universally experienced as helpful. 

In addition to health care services, participants talked about other organized sources of support that helped them stay safe. Participants described attending a variety of helpful groups, including groups for alcohol and drug recovery, parenting support, anger management, grief, and recovering from abuse. A common theme among all of these organized sources of support, both within and outside the health care system, is the opportunity to connect with other people (or animals) who were experienced as genuinely caring by the participant and who are available and willing to offer non-judgmental counsel and/or company during difficult times and at other times. 

#### 3.3.3. Self-Support

In addition to finding support from others, participants talked about the support they gave themselves to prevent or minimize suicide ideation. This self-support was expressed as behavior, cognition, and the desire to help others. Behavioral expressions of self-support was described by participants as keeping busy with activities and hobbies, exercise, engaging in spiritual activities (prayer, mantra, spending time in nature), and taking classes to increase knowledge and understanding. 

For several participants, self-support occurred by thinking about the impact their death by suicide would have on loved ones. Several participants described their awareness of the harm their suicide would cause family and friends as critical in preventing them from acting on suicidal thoughts. One participant who became suicidal after her son died by suicide said, “It hurt, and I thought, ‘Well, if I did it, then the rest of my family would hurt like I hurt.’ And I don’t want to do that to my family.” Another participant described a similar experience, “I thought of suicide twice [this past winter]. But then I said, ‘No [if I kill myself], I’m going to hurt my family. I’m going to hurt my children, I’m going to hurt many friends.’ And I’m like, ‘No, just because I have a disorder doesn’t mean I get to hurt everybody else and be selfish and do that to myself.’”

Participants also described using their experiences to help others as a way that they helped themselves. For several participants, being a survivor of suicide ideation and attempts gave their lives meaning and purpose that helped them to stay safe. Some participants felt that surviving suicidality had made them stronger and believed that sharing their stories could help others struggling with suicidality. One participant in her 20s stated: “If I survived all that, the whole chaos of my life, I’m here for a reason. And all the stuff I’ve went through, I can say [to a person contemplating suicide], ‘Well, I kind of know what you’re talking about.’ So, I could share my story, but also validate theirs.” “…That would be awesome to tell my story if that would help somebody else be out of that deep dark hole.”

## 4. Discussion

### 4.1. Main Findings

This qualitative study used an interpretive phenomenological approach to examine the narratives of 15 AN/AI adolescents and adults with a history of suicidality to understand what put them at risk and what helped them to stay alive during of high risk. Our data on participants’ suicide risk did not yield new insights, but they are consonant with previous studies of AN/AI suicide risk and help to contextualize participants’ narratives of help-seeking [7,42,43]. Our findings about participants’ help-seeking behavior underscore the central importance of both positive social connections and access to health services for people with suicide risk. Some social interactions were brief but impactful, such as the police officer that communicated his concern for one participant, while other meaningful interactions described were more long-term, such as the interaction with a trusted behavioral health professional. Importantly, these data highlight the power of social stigma in preventing or discouraging people from seeking help for suicidality, even from trusted adults or connected to formal crisis intervention services.

The sooner people sought and received help that they perceived as supportive, the shorter and less severe their suicidality. Some people did not seek help, due to stigma or lack of resources, until years after the onset of suicidality; these individuals tended to have multiple suicide attempts, more medical problems related and unrelated to attempts, and higher needs for ongoing services, such as case management or assisted living, to prevent suicide. Suicidality may present differently for individuals from varying backgrounds, including AN/AI people [44]. Research is needed to identify early expressions of suicidality and culturally appropriate models of intervention. Such interventions are needed to prevent and reduce social stigma and ensure that AN/AI individuals receive early intervention for suicide risk and ideation. 

Our study sought to understand the experience of people who wanted to kill themselves but decided, sometimes after multiple suicide attempts, to continue living. Understanding factors that protect people with suicide risk has the potential to inform suicide prevention efforts not only for AN/AI people but for other populations as well. Joiner’s interpersonal theory of suicidal behavior (ITS) posits what is necessary for a person to die by suicide. It proposes that suicide will not occur unless the individual experiences three psychological states [45]: thwarted belongingness, perceived burdensomeness, and capability of suicide. The first of these states, thwarted belongingness, relates to the fundamental need for social belonging. The experience of thwarted belonging was consistently evident in our participants’ narratives. Participants’ unanimously described lacking or being actually cast out of critical relationships or social roles and attributed their suicidality in some measure to these experiences. Conversely, they described experiences of acceptance and belonging that helped them to keep living. For example, several participants described AN culturally informed services such as Denaa’ Yeets in which someone called to check in on them, organized culturally relevant group activities, and took them places they might not be emotionally able to go on their own such to rivers for fishing outings. Others found acceptance from a family member or colleague who supported them. 

The second construct of ITS, perceived burdensomeness, postulates that the suicidal individual experiences his or her existence as being such a burden to others that it justifies the decision to end one’s life [46]. In perceived burdensomeness, the individual views their death as being more valuable to others than their life [46]. Our data show it is possible for people to perceive their death, particularly their death by suicide, as a greater burden to loved ones than their continued existence. Our participants expressed this concept multiple times and attributed it to keeping them alive even when they continued to experience thoughts of suicide. The awareness of how their death by suicide would impact their loved ones was what they said kept them from killing themselves during times of difficulty. This experience—of death becoming a greater perceived burden than life—became a critical protective factor among people with chronic suicidality. Clinical or educational interventions to foster this awareness could prevent or shorten the duration of chronic suicidality.

Our findings support and complement ITS with an explanation of why people choose to continue living, even despite ongoing, acute, and chronic suicidality. For several participants, the experience of recognizing and accepting that they were suicidal, learning and practicing skills to manage stress and painful emotions, and building a supportive network of people and places fostered a dedication to help others by telling their stories. Developing a deeper connection to others by sharing their experience of suicidality allowed them to transform a painful, personal experience into a compassionate act of helping others to heal. 

### 4.2. Implications for Health Systems

There are several key implications for health systems that aim to reduce suicide in populations they serve from these data. First, social stigma associated with suicide and mental illness prevented many participants in this study from seeking support. It may be beneficial for health systems to invest in efforts to de-stigmatize suicide and foster a culture of acceptance and understanding about suicidality. As suggested by Wexler et al. (2014), interventions where youth are provided an opportunity to contribute to the community rather than focusing on educating or increasing coping skills could promote resilience. Resiliency strategies are situated in and derived from community member’s experiences, and their physical and social environments shape the creation of meaning [47]. Such efforts could include public service campaigns to increase community-wide awareness of suicide risk factor or programs to train peer counselors and promote talking about suicide. Participants also suggested providing suicide prevention services, especially for young men, that are not specifically about suicide prevention but rather focused on wellness, and therefore do not stigmatize or label participants as being suicidal.

Additionally, health systems must commit to transforming suicide statistics into success stories by establishing strategic targets for reducing population suicide rates, evaluating current suicide prevention strategies and services and adjusting or overhauling these where deemed appropriate, and partnering with other health systems as needed to achieve these goals. For participants in this study, this required learning and practicing skills to mitigate stress and enhance resilience when managing difficult emotions. Just as individuals with suicide risk need new tools and practices, so do health systems and communities. Suicide prevention practices and policies can incorporate the lessons of lived experience. 

### 4.3. Implications for Practice

These findings have several key messages to inform service providers and policy makers. First, the importance of providing opportunities for suicidal people to learn new behaviors and skills was emphasized. Financial and personnel resources are needed to target emotional regulation, stress tolerance, interpersonal skills, and self-care skill building. 

Creating caring connections between patients and the health system should be a high priority. Primary care providers and behavioral health clinicians should seek and take into account their patients’ levels of belongingness, burdensomeness, and acquired capability (especially previous suicide attempts), in order to assess suicide risk and develop treatment to address core ITS constructs. 

Participants in this study found help to be effective only from individuals they perceived as genuinely caring, and they responded best to services that respected and reflected their cultural identities. The lived experience of participants in this study would not have been observed in an objective measure of protective factors. Participants in this study described a sense of responsibility to share their experience of suicidality with the purpose of helping others. Providers serving AN/AI communities may benefit from including lived experiences as a practice for treatment for suicide intervention and prevention services and policies. 

### 4.4. Strengths and Limitations

This study is among the first to report on lived experiences of suicidality among AN/AI people, which are necessary to understand how AN/AI people experience suicide risk and the experiences that contribute or hinder resilience to risk. While IPA requires smaller sample sizes to allow for the collection of in-depth data about lived experience and the complex contextual factors that shape it, the generalizability may be limited. This study was confined to AN/AI individuals in a single health system, providing in-depth insight into a population of interest while perhaps limiting the transferability of the findings to other populations. The inclusion criteria did not specify that participants must have had recent experiences of suicidality. The wide time range (6 months to more than a decade) of participants’ last experiences with suicidal thoughts or behaviors may have impacted their recall. While participants with a more recent experience may have had greater recall, participants with a more distant experience may have more generalized or refined memories of their experiences. Our sample was restricted to people who used health services, and did not include the experiences of individuals living with suicidality outside of a health system. Similarly, recruiting from within a health care system may have contributed to a selection bias for individuals with positive help-seeking experiences and health care services and an underreporting of negative experiences.

## 5. Conclusions

An interpretive phenomenological approach is ideal for examining lived experiences of suicidality and the social and cultural contexts in which they occur. The experiences of the AN/AI individuals in this study offer several insights for developing interventions that reduce suicide risk and promote resilience to suicide in AN/AI communities. Some findings can be generalized, while others are population specific due to considerations of history, geography, and culture. For the participants in this study, early intervention for preventing chronic suicidality, positive social connection, and perceiving one’s death as a greater burden to loved ones than one’s life helped to reduce suicide risk and contribute to resilience. These findings suggest that health systems and clinicians working with AN/AI people with suicide risk should work toward developing a heightened awareness of how suicide impacts family members and communities; and increasing suicidal individuals’ sense of social belonging and connectedness. Conversely, the role of social stigma as a barrier to seeking help for suicidality cannot be over-stated and must be prioritized by health systems and communities to reduce suicide risk. This study has important implications for health systems, service providers, and policy makers aiming to prevent suicide among AN/AI populations. Population- and interpersonal-level interventions were experienced as effective when grounded in AN/AI cultural frameworks. 
